# 3D map of the human corneal endothelial cell

**DOI:** 10.1038/srep29047

**Published:** 2016-07-06

**Authors:** Zhiguo He, Fabien Forest, Philippe Gain, Damien Rageade, Aurélien Bernard, Sophie Acquart, Michel Peoc’h, Dennis M. Defoe, Gilles Thuret

**Affiliations:** 1 Laboratory “Biology, Engineering, and Imaging of Corneal Graft”, BiiGC, EA2521, Faculty of Medicine, University of Saint Etienne, Saint Etienne, France; 2 Pathology Department, University Hospital, Saint-Etienne, France; 3 Eye Bank of St-Etienne, French Blood center, Saint-Etienne, France; 4 Department of Biomedical Sciences, Quillen College of Medicine, East Tennessee State University, Johnson City, TN 37614, USA; 5 Institut Universitaire de France, Paris, France

## Abstract

Corneal endothelial cells (CECs) are terminally differentiated cells, specialized in regulating corneal hydration and transparency. They are highly polarized flat cells that separate the cornea from the aqueous humor. Their apical surface, in contact with aqueous humor is hexagonal, whereas their basal surface is irregular. We characterized the structure of human CECs in 3D using confocal microscopy of immunostained whole corneas in which cells and their interrelationships remain intact. Hexagonality of the apical surface was maintained by the interaction between tight junctions and a submembraneous network of actomyosin, braced like a drum. Lateral membranes, which support enzymatic pumps, presented complex expansions resembling interdigitated foot processes at the basal surface. Using computer-aided design and drafting software, we obtained a first simplified 3D model of CECs. By comparing their expression with those in epithelial, stromal and trabecular corneal cells, we selected 9 structural or functional proteins for which 3D patterns were specific to CECs. This first 3D map aids our understanding of the morphologic and functional specificity of CECs and could be used as a reference for characterizing future cell therapy products destined to treat endothelial dysfunctions.

The endothelium is the innermost layer of the cornea, separating the corneal stroma from the liquid called aqueous humor that fills the anterior chamber. It is composed of flat cells of 5 μm thickness that form a uniform monolayer on an amorphous collagenous membrane (Descemet’s membrane). These cells cover the entire corneal concavity up to the trabecular meshwork, which is situated at the angle between the cornea and the iris. The endothelium plays several essential roles in corneal homeostasis. Besides regulating passage of nutrients and metabolic wastes to and from stromal keratocytes, its main role is the control of stromal hydration. The collagenous stroma is highly hydrophilic and, without the endothelium, rapidly swells and becomes opaque because edema disrupts the specific organization of collagen fibrils responsible for corneal transparency. Corneal endothelial cells (CECs) are terminally differentiated cells, equipped with different types of enzymatic pumps. These pumps create ionic gradients between the cornea and the aqueous humor, and are responsible for permanent extraction of water from the stroma^1^,^2^. Their situation on the only transparent tissue of the organism allows them to be observed non-invasively *in vivo*. Using specular microscopes -a routine ophthalmology device- CECs appear as polygonal cells, mostly hexagonal in healthy corneas. However, this feature only corresponds to the shape of the apical surface, where the cell is in contact with the aqueous humor. Because this imaging technique (specular microscopy) uses the 3–5% of light reflected by the interface between cell and water, this is the only surface accessible. At the opposite surface, in contact with Descemet’s membrane, CECs have an irregular shape that was described more than 30 years ago using transmission electron microscopy. However, because transmission electron microscopy only showed ultrastructural details, the exact 3D organization of the CEC has never been clearly described, and the structure-function relationships remain only partially understood. Recently, using immunolabeling techniques that we specifically optimized for whole corneas with intact endothelium^3^,^4^, and by studying proteins with restricted subcellular localizations, we obtained en face observations of CECs in their physiologic state. For instance, using labeling for NCAM, whose expression is restricted to lateral membranes, we revealed the global architecture formed by the complex basolateral membrane web with folds and interdigitations. In addition, we have used two Cre/Lox-dependent mouse strains that exhibit mosaic expression of cytosolic fluorescent proteins to visualize CEC morphologic complexity in this species^5^.

Worldwide, with one donor cornea available when 70 would be necessary, the scarcity of donor tissue is overwhelming^6^. Among the 185,000 corneas grafted yearly throughout the world, 38% concern endothelial dysfunctions. Such conditions can be efficiently treated by the selective replacement of the endothelial layer only, not the whole thickness graft necessary in other corneal blinding diseases^7^. While today this is achieved using a donor cornea cut into thin lamellae, tomorrow this selective graft could be performed using bioengineered products. Using CECs cultured *in vitro*, the deficient CECs could be replaced either by directly injecting new healthy cells behind the cornea, or through the construction of endothelial grafts by seeding new healthy cells onto a transparent thin carrier^8^,^9^. New methods for mass culturing of CECs from primary cultures or guided differentiation of stem cells are being developed. However, the bioengineered neo-endothelium must closely mimic the native endothelium if such therapeutic strategies are to be successful. That such a monolayer of cells is joined together by junctions may not be sufficient. Whatever the culture process adopted, the final product will have to contain only fully differentiated CECs. Identity and purity of the amplified cells are two critical quality attributes that will have to be verified before injection into the patient. Nevertheless, at present, there is no consensus agreement about the definition of CEC markers such as exists, for instance, for stem cells^10^. Recently, glypican, CD200, CD166 and membranous Prdx-6 have been proposed as specific CEC markers.

In the present work, using immunostaining on normal CECs of whole human corneas, we analyzed the subcellular localization of several structural and functional proteins (known or suspected to be expressed by CECs) in order to define their 3D map.

## Results

### Pre-selection of antibodies targeting corneal endothelial cells

Results of the screening among the 40 initial targets were summarized in Table 1. This provides, for each antibody, the best labeling obtained with either methanol or paraformaldehyde fixation. No labeling was found for proteins known to be absent in CECs. Immunolabeling quality of 8 proteins already described in human CECs (NSE, CD200, glypican, CLCN3, SLC4A4, VDAC3) or in animal CECs (aquaporin 1, CD44) was not satisfying, most of the time because of inconsistent staining, within the same tissue and between tissues. Nevertheless, only one commercial antibody was tested for each of these targets. Eighteen antibodies (for 17 proteins) matched our criteria for endothelial labeling quality: actin, myosin IIa, calnexin, CD166, connexin 43, COX IV, integrin α3β1, Na^+^/K^+^ ATPase, N-cadherin, NCAM, Tag-2A12/peroxiredoxin-6, α-tubulin, vimentin, ZO-1, histone H3, fibrillarin, and NUP98. There was no difference in staining intensity or in subcellular localization between freshly isolated corneas (body donation to science or from the operating theater), when compared with organ cultured corneas. We excluded the three nuclear proteins (histone H3, fibrillarin, and NUP98) for the next step of selection because they were ubiquitous proteins and were consequently not expected to be differentially expressed in other cell types.

### 3D mapping of protein expression

#### Apical surface: hexagonal

**Figure 1:**
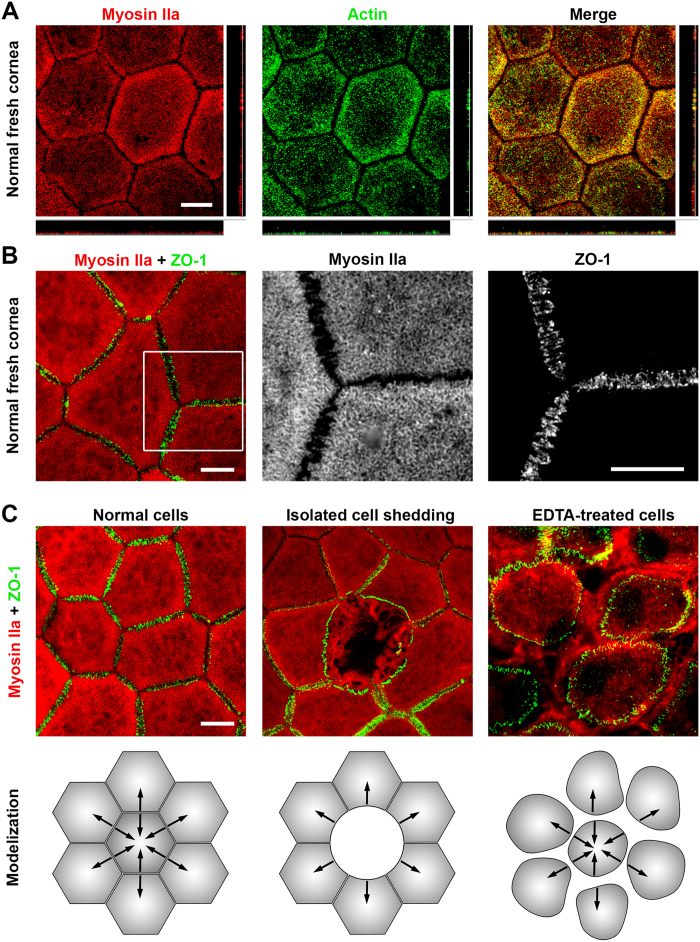
The shape of the apical surface of the corneal endothelial cells (CECs) depends on the actomyosin network and tight-junctions. (**A**) Myosin IIa and actin colocalized and formed a network just beneath the apical cell membrane. (Confocal microscopy, central cornea from a 16 years old patient with normal CECs and keratoconus) (**B**) The myosin network was perfectly aligned and connected with tight junctions, here highlighted by one of its components, ZO-1 (Epifluorescence microscopy, central cornea of a 68 years old patient with normal CECs and stromal hereditary dystrophy). (**C**) Illustration of the contractility of the actomyosin network in 3 situations. In normal CECs, forces between cells were balanced and responsible for straight borders. In the case of spontaneous isolated death, neighbor cells presented only one retracted border. The lateral cytoplasmic expansions became clearly visible in the area left empty by the dead cell. After treatment with ethylenediaminetetraacetic acid, which disrupted tight junctions, contraction of the network rounded the apical surface while leaving the lateral expansions almost intact. (Pair of corneas of a 66 years old donor with normal CECs).

**Figure 2:**
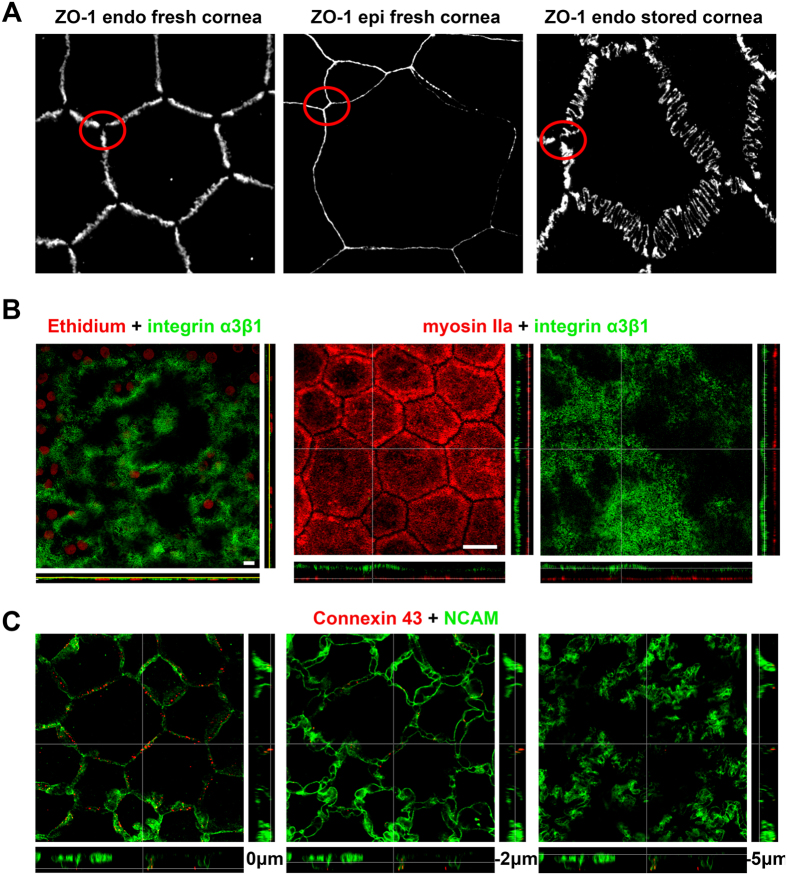
Additional features of human corneal endothelial cells (CECs). (**A**) Specificity of ZO-1 expression in CECs in fresh and stored corneas compared to superficial epithelial cells. The zig-zag pattern observed in a fresh cornea (65 years old patient with stromal dystrophy) was dramatically amplified in a cornea placed for 35 days in organ culture medium (74 years old donor with normal cornea). In both cases, ZO-1 remained absent at the Y junctions between cells (red circles), in contrast with the uniform distribution in epithelial cells. (**B**) Integrin α3β1 was almost evenly expressed in the basal membrane, following the slight ripples of the underlying Descemet’s membrane. Both samples were from 21 and 27 year-old patients, grafted for keratoconus. Scale bars = 10 μm. (**C**) Labeling of gap junctions (connexin 43) showed discontinuous points situated mainly in the subapical lateral membranes (revealed by NCAM); it could be found at intermediate levels but seldom at basal levels.

**Figure 3:**
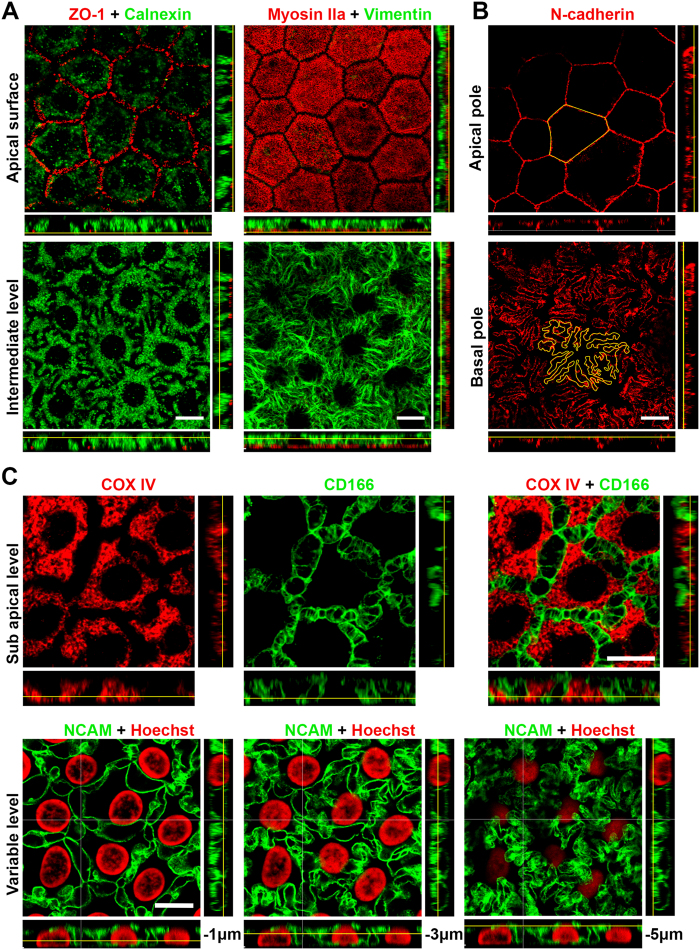
3D complexity of the lateral and basal cytoplasmic membranes of human corneal endothelial cells (CECs). (**A**) Labeling of calnexin in the endoplasmic reticulum (central cornea from a 48 years old patient with stromal lattice dystrophy) or labeling of the intermediate filament vimentin (central cornea from a 16 years old patient with keratoconus), highlight the lateral expansions of the cytoplasm, which create cell interdigitation. ZO-1 and myosin IIa were used to identify the apical surface. (**B**) The transmembraneous N-cadherin highlighted the opposing surfaces of CECs, showing maximal expansions of cytoplasmic membrane in contact with Descemet’s membrane, in contrast to the regular hexagonal shape of the apical surface. Cell contours were manually drawn (in yellow) to measure the cell perimeter (central cornea from a 16 years old patient with keratoconus). (**C**) Double-labeling of COX IV (mitochondrial network) and CD166 (lateral membranes) revealed the complexity of lateral membranes that delimited intercellular segmented spaces beginning immediately beneath the apical surface (central cornea from a 65 years old patient with stromal lattice dystrophy). The labeling of NCAM, expressed exclusively in the lateral membranes, showed, at 3 different levels, the increasing tortuosity of the membrane expansions toward Descemet’s membrane (central cornea from a 36 years old patient with keratoconus. All patients of this panel had a normal corneal endothelium).

Actin and myosin IIA colocalized and were located exclusively just beneath the apical plasma membrane, forming a thin layer well separated from the rest of the cytoplasm, which was filled with vimentin intermediate filaments. Zonula occludens-1 (ZO-1), a submembraneous protein of the tight junction complex universally used to demonstrate the hexagonal shape of CECs, was found at exactly the same level as the actomyosin network and directly connected with it, as expected (Fig. 1A,B). ZO-1 was always distributed following a zig-zag line that was amplified in organ-cultured corneas (Fig. 2). Contrary to the basal surface (labeled with anti-integrin α3β1, Fig. 2B), the actomyosin network appeared particularly regular and flat, reflecting a surface tension. In addition, observation of few isolated dying cells randomly distributed in several fresh corneal buttons showed disruption of this network and shrinkage of the corresponding ZO-1 lines (Fig. 1C). Finally, immunostaining performed after pharmacological disruption of tight junctions with the Ca^2+^ chelating agent Ethylenediaminetetraacetic acid (EDTA) showed a similar but amplified phenomenon. In the latter case, there was total loss of hexagonal shape caused by shrinkage of the actomyosin network bound to ZO-1 (Fig. 1C). These findings are further evidence that a tension in the apical actomyosin cytoskeleton is responsible for the straight borders and geometric hexagonal shape of the apical surface of CECs. N-cadherin, a calcium-dependent transmembrane protein also implicated in cell-cell adherence, was localized in the lateral membrane immediately under the apical surface, thus highlighting the hexagonal shape of CECs similarly to ZO-1 (Fig. 3B). However, N-cadherin was also found at greater depths (see below). Gap junctions highlighted by labeling of connexin 43 presented as discontinuous points situated mainly at subapical lateral membranes (Fig. 2C). They were also found at medium levels, but seldom at basal levels.

#### Laterobasal web: complex cellular interdigitations

Different cytoplasmic markers highlighted a non-hexagonal, irregular flower shape with increasing complexity toward the basal surface. For instance, staining for calnexin, a marker for the endoplasmic reticulum, or vimentin, an intermediate filament, clearly revealed the presence of cellular interdigitations that become more numerous and longer toward the basal surface (Fig. 3A). N-cadherin, present under the apical membrane, was also found expressed at the other extremity of the lateral membrane in contact with the underlying Descemet’s membrane. Staining of N-cadherin therefore perfectly outlined cell boundaries at both cell surfaces. We measured both perimeters in 10 cells of a fresh cornea (16 year old patient with a keratoconus, ECD of 3200 cells/mm^2^) and found a basal/apical ratio of 5.6+/−0.3. Areas of apical and basal surfaces were similar, respectively 312 ± 80 and 307 ± 56 μm^2^ (Mean ± SD, P = 0.959, Wilcoxon signed-rank test for paired data) (Fig. 3B). We found 4 other proteins in which expression was restricted to the lateral membranes: NCAM, CD166, Na^+^/K^+^ ATPase and peroxiredoxin-6 (Prdx-6) (Table 1). Continuous staining from the apex to the basal surface confirmed the progressive increase of the membrane expansions described above. In addition, thanks to the accuracy of the labeling for NCAM or CD166, intercellular spaces delimitated by lateral membrane folds were constantly visible along the whole height of the cell (Fig. 3C and Supplementary movie S1). Integrin α3β1 was the only protein found exclusively at the basal surface, forming an almost homogenous layer that follows the slightly bumpy surface of Descemet’s membrane (Fig. 2B).

### Endothelial-specific 3D-mapping of proteins

**Figure 4:**
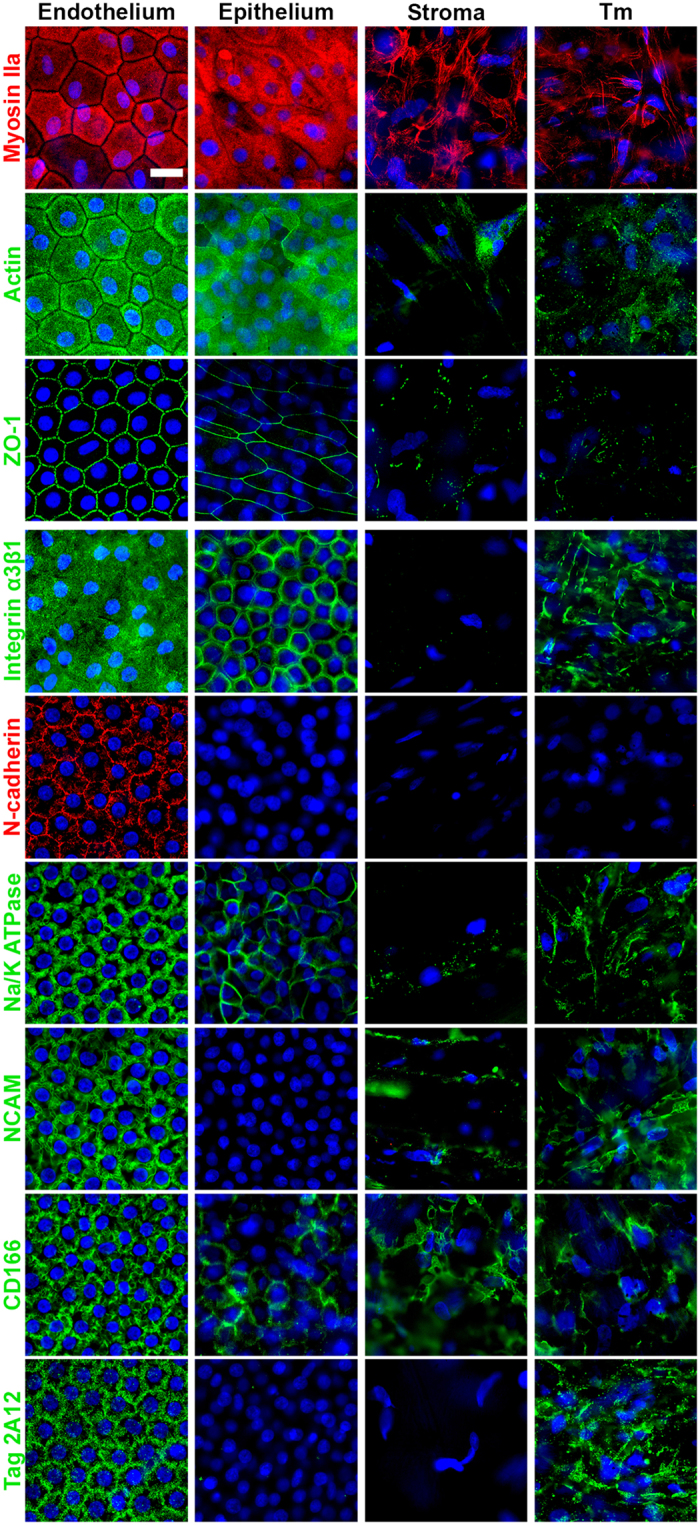
Biomarkers for human corneal endothelial cells (CECs). Nine structural or functional proteins were selected because of their specific staining pattern and subcellular location in CECs compared to corneal epithelial, stromal and trabecular meshwork (TM) cells. Except for N-cadherin, none of them was expressed only in CECs, but they all had a distinct pattern specific for CECs. Nuclei were counterstained by Hoechst 33342 (in blue). Scale bar 20 μm.

With the notable exception of N-cadherin, none of the proteins tested was specific for CECs. For instance, other corneal cells expressed ZO-1 and Na^+^/K^+^ ATPase, even though these are commonly used to identify CECs. Also, Prdx-6 and CD166 have recently been described as new potential markers for CECs. Nevertheless among the 14 preselected targets, we selected 9 structural and functional proteins whose subcellular localization had patterns found only in CECs and not in corneal epithelial cells, keratocytes or trabecular endothelial cells (Fig. 4). They could therefore be considered as hallmarks of CECs. In CECs, ZO-1 labeling was specifically distributed along a jigsaw pattern interrupted at the Y junction between 3 cells, contrary to the superficial epithelial cells where it formed a continuous regular straight line. The width of the zig-zag varied between corneas, short in fresh corneas and elongated in stored corneas, being an indicator of stress (Fig. 2A). The submembranous network of actin and myosin IIa, exclusively located at the apical surface and applying tension to the plasma membrane, did not exist in the other cells, in which both proteins only filled the cytoplasm. This was the case even in epithelial cells, which are nevertheless also polarized. The complex lateral membrane organization was also remarkable. Beginning immediately beneath the apical surface, the finger-like processes progressively expanded toward Descemet’s membrane, to form a complex network resembling the interdigitating foot processes of glomerular podocytes, without any equivalent in the 3 other corneal cell types. Lateral membranes were revealed, with almost similar staining patterns, by 5 antibodies targeting Na^+^/K^+^ ATPase, CD166, Prdx-6, NCAM, and N-cadherin. N-cadherin was the only marker found exclusively in CECs. Polarization of integrin α3β1 at the basal surface was found only in CECs, whereas it was located at cell-to-cell junctions in epithelial cells, absent in keratocytes and filled the whole cytoplasm or the whole plasma membrane of trabecular meshwork cells.

### Simplified 3D model of human corneal endothelial cells

**Figure 5:**
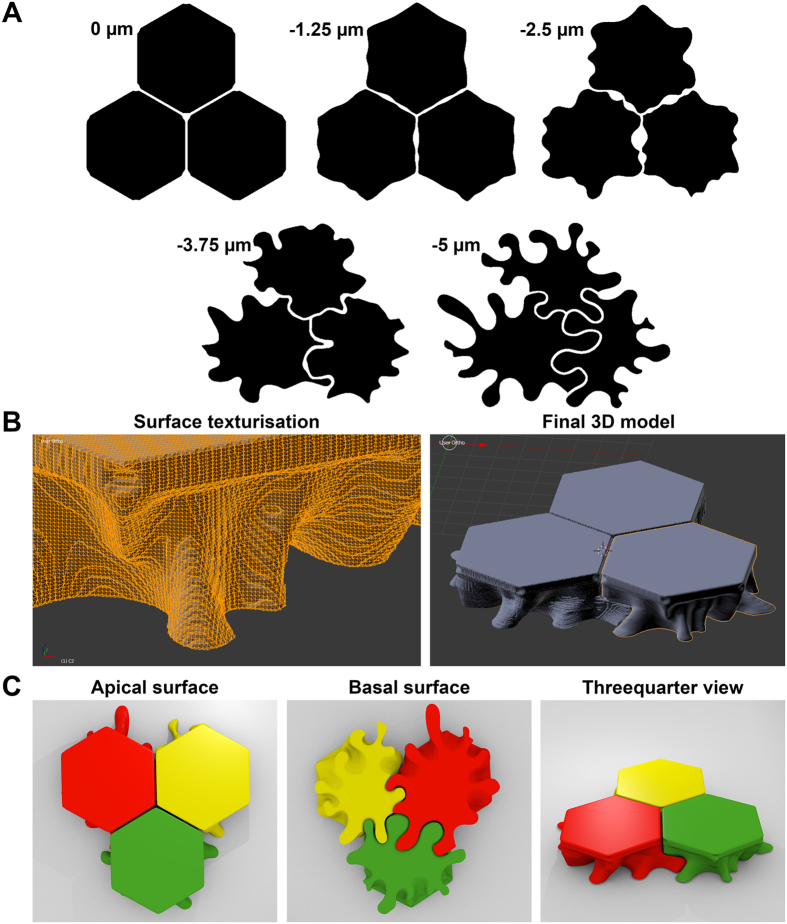
3D reconstruction of human corneal endothelial cells (CECs). (**A**) Five layers uniformly distributed over the CEC height were schematized using information provided by confocal images of membrane proteins. (**B**) Example of surface smoothing using the freeware blender and final 3D reconstruction of 3 neighbor CECs. (**C**) Different view of 3D printed cells for pedagogic purpose. The simplified model is consistent with the characteristics of cell-cell junctions: straight borders with holes at the Y junctions at the apical surface, interconnected intercellular spaces between cells, tightly joined interdigitating foot processes at the basal surface. Only the number of membrane folds and interdigitations might have been underestimated (see N-cadherin Fig. 2B).

The CADD-reconstructed CECs took account of the features described above for cytoplasmic membranes (Fig. 5). Virtual cross sections in any plane allowed visualization of cell-cell interactions. The interruption of the apical zonula occludens at the Y junction between 3 cells was in continuity with the intercellular spaces delimited by lateral membranes folds. The area of the lateral membranes of three cells subjected to simplified modeling was approximately 1.6 times higher than if the CECs had vertical straight hexagonal membranes. The three 3D-printed cells could be easily separated to show the lateral membrane expansion and intercellular spaces.

## Discussion

Using immunostaining protocols optimized for labeling CECs of whole human corneas, in combination with confocal microscopy, we established the 3D expression map of several structural and functional proteins in this cell type. Our work demonstrates the three-dimensional complexity of a cell that is only five micrometers thick. This is a point that is often ignored, as CECs are usually seen as flat, regular hexagons resembling terracotta tiles. We showed that the subcellular location of several proteins is a hallmark of the polarization of CECs that can be used as a specific marker to distinguish these cells from the other corneal cell types. In addition we identified N-cadherin as a specific CEC marker within the cornea.

The CECs have been extensively studied in the past where their role in maintaining corneal transparency has been clarified. The main components of CECs are known^11^,^12^,^13^,^14^,^15^,^16^ and their physiology is being modeled^1^,^2^. Yet, our work provides new insights into the structure-function relationships. Our observations indicate that an actomyosin network that is under tension between tight junction complexes maintains the hexagonal shape of the apical surface. This tension also explains the low pleiomorphism of CECs, as all cells are being initially submitted to the same tension. This apical structure may constitute a rigid lid protecting the cell against intraocular pressure (IOP), a function to which other, cytoskeletal proteins such as vimentin, which fills the cytoplasm, also contribute. Vimentin is known to protect cells against compressive stress^17^. As IOP is an important determinant of corneal physiology^18^, CECs are likely to possess specific structures that are related to their constant exposure to this physical stimulus. In addition, this contractile apical network may also be damaged by non-physiologic stimuli, like the rapid formation of posterior folds in stored corneas. Dead CECs predominantly observed at the top of these folds may result from an excess stress applied to the actomyosin molecules. Recently, we showed that the organization of peripheral CECs in centripetal rows and furrows imprinted in the peripheral Descemet’s membrane suggested a continuous, extremely slow migration of CECs toward the corneal center throughout life^19^. Whether the contractility of the actomyosin apical network may contribute or not to this migration remains to be investigated.

We also clarified the complex structure of the lateral membranes and propose a first quantification of the increase in area resulting from the formation of folds and interdigitating foot processes. This tortured shape allows multiplying by at least 1.6 the enzymatic pumping sites in a cell that is only 5 μm thick. Note that this may be an underestimation resulting from the need to simplify the first model designed for 3D printing. Other confocal analyses like the one we recently described on transgenic mice with fluorescent CECs may help enrich the 3D model^5^. The use of super-resolution microscopy may also be instrumental in increasing the accuracy of reconstruction. Nevertheless, the present 3D analysis was sufficient to improve our representation of the complex network of intercellular spaces in relation to the discontinuity present in tight junctions at Y junctions between 3 cells. These spaces are not present at the basal surface (as shown by the N-cadherin labeling), indicating that water may cross the basal membrane, enter the CEC and be excreted by the lateral membranes. These spaces may also explain how CECs of organ-cultured corneas are made visible under a transmitted light microscope in eye banks. Incubation with 0.9% sodium chloride with 1.8% sucrose temporarily alters the efflux of water, which then accumulates in these spaces. The dilated spaces filled with water may induce local modification of the refractive index and increase local contrast, making cell contours visible^20^.

The study of CECs is experiencing renewed interest since the development of new surgical techniques, called endothelial keratoplasty, which allow selective replacement of the endothelium in patients suffering from endothelial insufficiency^21^. In parallel, efforts to develop substitutes for donor corneas now bring up the possibility that stable mass cultures of CECs (for review, see Peh *et al*. 2011) will replace native tissue for future endothelial cell therapy^8^,^9^. Proofs of concept have been obtained in animals^22^,^23^. Despite numerous attempts to identify a unique antigen specific for the normal native CECs, there is no universal agreement about the specificity of these antigens. Using a different approach, we demonstrate that the native CEC presents a unique 3D mapping of several structural and functional proteins that are found neither in corneal epithelial cells, nor in stromal keratocytes and cells of the trabecular meshwork, the 3 other corneal cell types that may potentially contaminate CEC cultures. The correct subcellular location of these proteins is likely to be characteristic of the fully polarized CECs, that is, CECs forming a interconnected mosaic at the surface of a cornea in a physiologic or nearly physiologic environment (comprising for certain, nutrients and growth factors, cell cycle inhibitors and, more speculatively, the role of the intraocular pressure, light and temperature). It is unlikely that CECs proliferating *in vitro* will present the same 3D mapping of proteins as the native cells; therefore, quality controls will have to be performed on the final product, be it the reconstituted neo-endothelium (in case of a bioengineered graft) or the stabilized cells (in the case of a cell suspension). To summarize, a fully polarized CEC is defined by: 1) an hexagonal apical surface well characterized by ZO-1, actin and myosin IIa forming a thin network. Organization and/or maturation of this network might be dependent on the existence of an IOP. This point requires further investigations as, up to now, culture systems do not integrate a pressure gradient; 2) lateral membrane expansions increasing in complexity and forming interdigitating foot processes in contact with Descemet’s membrane. They contain NCAM, CD166, Na^+^/K^+^ ATPase, Prdx-6 and N-cadherin and present as flower-shaped cells under the epifluorescence microscope; 3) a basal surface expressing integrin α3β1. Interestingly, ligands of integrin α3β1, such as laminin-5A (-3A32)^24^, laminin-511, and laminin-521^25^ constitute efficient coating substances that improve the yield of *in vitro* CEC cultures. In addition to this triad of characteristics, expression of vimentin, although having no specific 3D pattern, can also be used, because it is a marker, not expressed in epithelial cells.

The case of N-cadherin is interesting because it was the only one of all the targets that was found exclusively in CECs. It could therefore be used to identify and sort cells during culture processes based on primary cultures of CECs. Furthermore, N-cadherin is expressed by neural crest cells^26^ and mesenchymal stem cells^27^, which might be the alternative stem cell sources for endothelial bioengineering^28^.

In conclusion, our study reveals new aspects of the cellular biology of human CECs that are essential for corneal transparency and, thus, one of the key targets in the treatment of corneal blindness. Our study establishes the first 3D map of proteins implicated in the polarity of mature CECs. It provides new insights into the structure-function relationships and suggests new markers to fully characterize the future advanced therapy products obtained during endothelial bioengineering processes destined to replace donor corneas.

## Materials and Methods

### Human corneas and ethics

Stored and fresh (un-stored by definition) human corneas were used. Sixty organ cultured corneas with mean donor age of 76 ± 12 (min 45, max 93) years were used to test a large of number of antibodies, in order to preselect the antibodies of interest. Stored corneas were obtained from the Eye Bank of Saint-Etienne and initially stored in 100 mL organ culture medium (CorneaMax, Eurobio, Les Ulis, France) at 31 °C for 21 ± 8 (8, 35) days. They were not suitable for clinical use because of serological non-conformity, but their endothelium was healthy (verified by the eye bank’s technicians). Fresh corneas were obtained from two sources. The first one was the central corneal buttons trephined during corneal grafts from patients presenting with a keratoconus or an hereditary stromal dystrophy frequent in our region. Both corneal diseases do not affect the endothelium. The mean age was 25 ± 9 (15, 36) years for the 12 patients with keratoconus and 58 ± 9 (40, 68) years for the 9 patients with lattice stromal dystrophy. Immediately after excision, the cornea was immersed in balanced salt solution (Alcon, Rueil Malmaison, France) to avoid desiccation. Time between corneal excision and fixation was about 30 minutes. These corneas were therefore as close as possible to *in vivo* conditions. These tissues are surgical waste and collected as per the usual protocol in force in our University Hospital and by presumed consent pursuant to the written information given to all admitted patients. This protocol was written by our Hospital’s commission for clinical research and innovation and accepted by the local ethics committee (CPP Sud Est I, CHU Saint Etienne, Saint Etienne, France, IRB00010220). The second source of fresh corneas was body donation for Science. Each donor volunteered their body and gave written consent to the Laboratory of Anatomy. Nineteen corneas were immediately fixed after dissection from the eyeballs and the mean donor age was 79 ± 9 (63, 91) years. The time between death and retrieval on refrigerated bodies was 13 ± 8 (3, 24) hours. Handling of donor tissues adhered to the tenets of the Declaration of Helsinki of 1975 and its 1983 revision in protecting donor confidentiality.

### Target proteins and antibodies

Because of the variable quality of the commercial antibodies^29^, the peculiarity of the immunostaining technique on flat mounted whole cornea, and the limited number of good quality human corneas available for research, we first preselected a list of 42 structural and functional proteins of interest that could potentially either highlight cell structure or be reliable markers for CECs. They were classified into four categories: 1) known markers of CECs: three proteins which have been universally used for years to define human CECs (ZO-1^11^, Na^+^/K^+^ ATPase^30^, Neuron specific enolase (NSE)^31^) and four, more recently described as CECs markers (CD200, Glypican 4^32^, CD166 (a member of the immunoglobulin superfamily of proteins)^14^, and peroxiredoxin-6 (Prdx-6/antibody Tag-2A12, an antioxidant enzyme)^33^); 2) proteins of the cytoskeleton. Most of these were already described in human CECs: the microfilaments of actin, myosin IIa^12^, the microtubules of tubulin^11^, the intermediate filaments vimentin^34^, cytokeratins CK 7, 18, 19^12^, and nestin (in foetal CECs only)^35^; 3) specific markers of organelles present in most nucleated cells: calnexin (endoplasmic reticulum), COXIV (mitochondria), CENP-A (nucleus, centromeres), fibrillarin (nucleolus), histone H3 (nucleosome), LC3B (autophagosomes), NUP98 (nuclear pore), Rab5 (endosome); 4) membrane proteins for which expression was previously reported in human CECs (connexin 43^15^, NCAM^13^, integrin β5^36^, N-Cadherin^16^, integrin a3b1^24^, and CLCN3, SLC4A4, VDAC3^37^) or in animal CECs (aquaporin 1^38^ and CD44^39^). Seven proteins served as negative controls: 3 cytokeratins (CK3/12, 5/6, 20), which are known markers of the corneal epithelial cell and described in human CECs only in pathological cases^40^, membrane markers for vascular endothelial cells (PECAM-1), for corneal epithelium (E-cadherin), and for keratocytes (CD34)^41^, and α-SMA as a marker for conjunctival epithelium that has been described only in monkey CECs^42^. The detailed list of the 47 corresponding antibodies is provided in Table 1. Three criteria were used for selecting antibodies for further investigations: staining intensity (+++ strong; ++ moderate; + faint; −negative), staining homogeneity (staining of all ECs, yes or no), and staining with a clear and coherent subcellular localization.

### Immunostaining on flat-mounted whole cornea

The immunostaining of flat-mounted whole corneas was specially optimized for the analysis of protein expression in intact CECs in this tissue^3^,^4^. Briefly, human corneas were fixed in pure methanol or 0.5% paraformaldehyde (PFA) for 30 minutes. They were then cut into six or eight pie-shaped wedges to increase the number of experiments while saving rare tissues. Permeabilization with 1% Triton 100-X was required for PFA-fixed corneas. Cornea rehydration was done in PBS at RT for 5 minutes for methanol-fixed corneas. The non-specific binding sites were then blocked by incubation of corneas in PBS containing 2% heat-inactivated goat serum and 2% bovine serum albumin (BSA) for 30 minutes at 37 °C. The primary antibodies were diluted at 1/200 in the blocking buffer and incubated with corneas for 1 hour at 37 °C. The secondary antibodies were diluted at 1/500 in the blocking buffer and incubated with corneas for 45 minutes at 37 °C. These consisted of Alexa Fluor 488 goat anti-mouse (A-11001, Invitrogen) and/or Alexa Fluor 555 goat anti-rabbit (A-21429, Invitrogen). Nuclei were finally counterstained with 5 μg/mL Hoechst 33342 (Sigma) in PBS at RT for 5 min for epifluorescence microscopy or with 4 μM ethidium homodimer (FP-25810A, Fluoprobes) in PBS at RT for 5 minutes for confocal microscopy. Three rinses in PBS were performed between all steps, except between saturation of non-specific protein binding sites and incubation with primary antibody. The corneal pieces were finally placed on glass slides, covered with Vectashield Mounting Medium (H-1000, Vector Laboratories) and gently flattened using a large glass coverslip retained immobilized by adhesive tape. Experiments were done at RT unless otherwise stated. In order to analyze morphologic changes after disruption of the tight junctions, other samples were incubated with a saturated solution of ethylenediaminetetraacetic acid (EDTA) in PBS without Ca^2+^ or Mg^2+^ for two and a half minutes at RT. Corneas were then immediately fixed in 0.5% PFA for immunostaining as described above.

For all labeling, the images were first acquired using an epifluorescence inverted microscope IX81 (Olympus, Tokyo, Japan) equipped with the Cell^P imaging software (Soft Imaging System GmbH, Munster, Germany). The objectives were UPIanFL N, 4x/0,132; CPIanFL N, 10x/0,3 phc; UPIan PL N60x/1,25 oil iris, and PIanSApo100x/1,40 oil (Olympus). Secondly, for selected antibodies, in order to highlight their spatial organization and subcellular distributions, confocal stacks were obtained by using a FLUOVIEW FV1200 laser scanning confocal microscope (Olympus, Tokyo, Japan) equipped with the FV10-ASW4.1 imaging software. The objective was an Olympus UPlanSApo 60x/1.35 Oil ∞/0.17/FN26.5.

**Figure 6:**
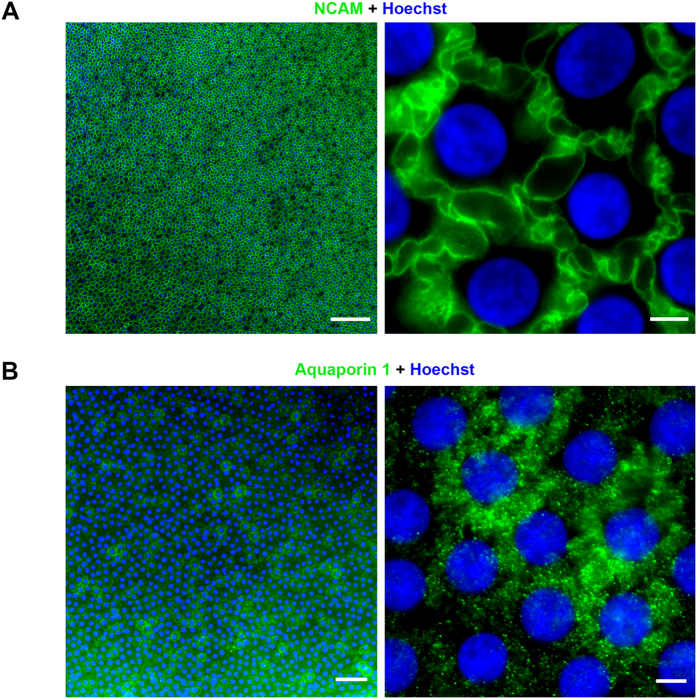
Grading of immunostaining of human corneal endothelial cells (CECs) using epifluorescence microscopy. (**A**) Labeling of Neural Cell Adhesion Molecule (NCAM) with the MAB24081 from R&D systems gave a bright homogeneous staining of all CECs and had a clear subcellular localization in the lateral membranes. Left panel, low magnification, scale bar = 200 μm. Right panel, high magnification, scale bar = 5 μm. (Thirty-six years old patient with a keratoconus). (**B**) Labeling of aquaporin 1 with the ab9566 from Abcam was too heterogeneous and had no clear subcellular localization to be considered as reliable. Left panel, low magnification, scale bar = 50 μm. Right panel, high magnification, scale bar = 5 μm. (Sixteen years old patient with a keratoconus).

The selection and optimization steps were performed for each antibody using the two different fixatives (methanol and 0.5% PFA)^3^,^4^ on two different organ-cultured corneas (more numerous) and one fresh cornea. The first step was aimed at selecting only antibodies that demonstrated a reliable staining, defined by an homogeneous bright staining of the targeted protein in all CECs and a clear subcellular location (Fig. 6). Except for the extreme periphery of the endothelium, for which we demonstrated that CECs were less differentiated^19^, the rest of the endothelium was considered to constitute an homogeneous population of cells. We chose labelings that were clearly restricted to distinct cell compartments and/or even more precisely restricted to a specific location within a compartment (for instance proteins located only in the lateral cytoplasmic membranes). In the case of absence of staining for proteins known or very likely to be expressed by CECs, a second antibody targeting the same protein was assessed. The selected antibodies were tested again on whole fresh corneas, using optimized parameters such as fixative and temperature, in order to approach as closely as possible the physiological state and to avoid potential artifacts caused by prolonged death to retrieval time and corneal storage. Using confocal images of the proteins described above, we established a 3D map of CECs from the apical to the basal surface. In a second step, their specificity for CECs was further tested.

### Selection of specific endothelial markers

Given the 3D mapping of numerous proteins in CECs, we hypothesized that a select subgroup of proteins could be sufficient to fully characterize CECs in term of protein expression within a particular physiological location. In order to select several potential markers with an endothelium-specific 3D mapping, we assessed the expression of 14 proteins in the 3 other corneal cell types *in situ* or on whole corneas. In this step, we considered as specific, any markers that were either absent in the 3 other corneal cell types or expressed in other cell types but with a distinct subcellular localization in CECs. For immunostaining of epithelial cells on whole corneas, we previously showed that the same protocols as described above were suitable^3^. For keratocytes, we simply observed the keratocytes visible on the edges of the cut corneal pieces. The radial cuts were deliberately not orthogonal to the surface but beveled, in order to increase the area of the exposed stroma. Immunolabeling of the four cell types was therefore performed simultaneously. All labeling was repeated at least two times in two different organ-cultured corneas and in two different fresh corneas.

### 3D reconstruction of the human corneal endothelial cell

Ultimately, we reconstructed a 3D model of 3 neighbor CECs using computer-aided design and drafting (CADD) software packages. Given the complexity of distinguishing the individual contours of two neighbor cells when lateral membranes were labeled, we combined the information provided by confocal images acquired at 5 depths for 3 markers (NCAM, N-cadherin and Na^+^/K^+^ ATPase) on 4 corneas (chosen with similar endothelial cell density to facilitate comparisons). We extrapolated the most likely contours to manually draw a simplified model of 3 adjacent cells. For each of the 3 cells, we manually sketched five sections of CECs on five horizontal scans. The first scan was located at the apical surface, corresponding to the belt of tight-junctions belt and the last one was where the cell contacted Descemet’s membrane. The sketches were digitized and extrapolated plane by plane (40 planes per cell) with Solidworks 2015 (Vélizy Villacoublay, France). Using the freeware ImageJ, the stack of 40 planes was extrapolated to 100 planes. The resolution was then decreased to 300 × 300 pixels per cell to facilitate smoothing without loosing cell details. Images were converted to binary and the 3D model was reconstructed using the 3Dviewer plugin and exported in the .stl format file. A third software package, Blender (Stichting Blender Foundation, Amsterdam, the Netherlands, freeware downloadable at https://www.blender.org/download/) was used to smooth the shape. Finally, for pedagogical purposes, 3 adjacent cells were printed using a 3D printer (ProJet 3510 HD, 3D system, Rock Hill, SC), using the highest resolution (16 μm per layer) and VisiJet M3-X as a material. As necessary, the design of the lateral expansions was slightly modified to allow disassembly of the 3 cells.

## Additional Information

**How to cite this article**: He, Z. *et al*. 3D map of the human corneal endothelial cell. *Sci. Rep*. **6**, 29047; doi: 10.1038/srep29047 (2016).

## Supplementary Material

Supplementary Information

Supplementary Movie S1

## Figures and Tables

**Table 1:** List of antibodies tested on human corneal endothelial cells (CECs) using an optimized whole mount technique and summary of their results.

Cat	Target Protein	Source	Laboratory	Reference	Fixatives	Staining intensity	Staining homogeneity
Known corneal endothelial markers§	CD166	mouse	*BD Pharmingen*, San Diego, CA	559260	M	+++	yes
	Na^+^/K^+^ATPase	mouse	Millipore, Billerica, MA	05–369	M	+++	yes
	Na^+^/K^+^ ATPase	mouse	Abcam, Cambridge, UK	ab7671	PFA	++	yes
	ZO-1	rabbit	Zymed, Carlsbad, CA	40–2200	PFA/M	+++	yes
	ZO-1	mouse	Zymed	33–9100	PFA/M	+++	yes
	Prdx-6/Tag 2A12*	rabbit	Bioprocessing Technology Institute, Singapore	/	M	+++	yes
	NSE	mouse	Leica Biosystems Newcastle, UK	Pa 0435	M	++	no
	NSE	mouse	Abcam	ab16808	M	+	no
	Na^+^/K^+^ ATPase	rabbit	GeneTex, Irvine, CA	GTX 113390	PFA/M	+	no
	CD200	mouse	Abcam	ab23552	PFA	+	no
	Glypican 4	mouse	Abcam	ab100–843	PFA	+	no
Cytoskeleton	Actin	mouse	Sigma, St. Louis, MO	A4700	M	+++	yes
	Myosin IIa	rabbit	Abcam	ab24762	PFA	+++	yes
	Tubulin α	mouse	Abcam	ab7291	PFA	+++	yes
	Vimentin	mouse	Abcam	ab8978	PFA	+++	yes
	Vimentin	mouse	Dako, Glostrup, Denmark	M0725	PFA	++	yes
	CK7	mouse	Dako	M7018	PFA	+++	no
	CK18	mouse	Dako	M7010	PFA/M	+++	no
	CK19	mouse	Dako	M7888	PFA	−	
	Tubulin α	rabbit	Cell signaling Technology, Danvers, MA	#2125	PFA/M	−	
	Nestin	rabbit	Abcam	ab92391	PFA	−	
Organelles	Calnexin	mouse	Abcam	ab31290	PFA	+++	yes
	COX IV	rabbit	Cell Signaling	#4850	M	+++	yes
	Fibrillarin	rabbit	Cell Signaling	#2639	M	+++	yes
	Histon H3	rabbit	Abcam	ab1791–100	M	+++	yes
	NUP98	rabbit	Cell Signaling	#2598	M	+++	yes
	LC3B	rabbit	Cell Signaling	#3868	PFA	++	no
	CENP-A	rabbit	Cell Signaling	#2186	PFA/M	++	no
	Rab5	rabbit	Cell Signaling	#3547	PFA	+	No
	Calnexin	rabbit	Cell Signaling	#2679	M/PFA	−	
Membrane proteins	N-cadherin	rabbit	Cell Signaling	#13116	PFA	+++	yes
	NCAM	mouse	R&D systems, Minneapolis, MN	MAB24081	M	+++	yes
	connexin 43	mouse	Cell Signaling	#3512	M	+++	yes
	Integrin α3β1	mouse	Dako	M0608	PFA	+++	yes
	Integrin b5	rabbit	Millipore	AB1926	PFA	+	yes
	VDAC3	rabbit	Santa Cruz	sc-292328	M	++	yes
	Aquaporin 1	mouse	Abcam	ab9566	PFA	++	no
	CLCN3	rabbit	Abcam	ab28736	M	+	no
	SLC4A4	rabbit	Abcam	ab56215	M	+	no
	CD44	mouse	Abcam	Ab6124	M	+	no
Negative controls	CK20	mouse	Dako	M7019	PFA	−	
	CK3/12	mouse	Abcam	ab68–260	PFA	−	
	CK5/6	mouse	Dako	M7237	PFA	−	
	α-SMA	mouse	Abcam	ab7817	PFA	−	
	PECAM-1	mouse	Santa Cruz	Sc-20071	PFA/M	−	
	CD34	mouse	Dako	IM0786	PFA	−	
	E-cadherin	mouse	Zymed	33–4000	M	−	

For Na^+^/K^+^ ATPase, NSE, vimentin, tubulin α, and calnexin, a first antibody did not provide the expected result (in conformity either with the literature or for ubiquitous proteins); consequently a second one was assessed. Eighteen antibodies (in bold) corresponded to the quality criteria. Excluding fibrillarin, histone H3, and NUP98 (ubiquitous nuclear proteins used as positive controls for the immunostaining technique), 15 antibodies (14 proteins) were selected. Seven target proteins known to be absent in CECs served as negative controls. Other negative or heterogeneous results must be taken with caution: they meant that the tested antibody did not react with CECs or with all CECs, not necessarily that CECs did not express or express homogeneously the corresponding protein. Lack of specificity, affinity or avidity could be involved. Due to the limited amount of human material, we could not always assess other antibodies. M - methanol; PFA-paraformaldehyde.
