# Supplementary material for: 3D map of the human corneal endothelial cell
Source: Sci Rep. 2016 Jul 6;6:29047. doi: 10.1038/srep29047 (PMC4933894; doi:10.1038/srep29047)
Supplement: Supplementary Information [file srep29047-s1.doc]

**3D map of the human corneal endothelial cell**

**Running Title: 3D map of the corneal endothelial cell**

Zhiguo He^1^, Fabien Forest^1,2^, Philippe Gain^1^, Damien Rageade^1^, Aurélien Bernard^1^, Sophie Acquart^3^, Michel Peoc’h^1,2^, Dennis M. Defoe^4^, Gilles Thuret^1,5^

**Legend for supplementary movie**

**Movie S1. Morphological changes in Z of lateral membranes of human corneal endothelial cells (CECs).** Lateral membranes were visualized using the immunostaining of NCAM (green). Nuclei were counterstained by ethidium bromide (red). Forty slices in Z (0.2µm/slice) were obtained by confocal microscopy and were converted into a movie using ImageJ. Central cornea from a 36 years old patient with keratoconus.
